# Emergence of Polycystic Neotropical Echinococcosis

**DOI:** 10.3201/eid1402.070742

**Published:** 2008-02

**Authors:** Dennis Tappe, August Stich, Matthias Frosch

**Affiliations:** * University of Würzburg, Würzburg, Germany; † Medical Mission Hospital, Würzburg, Germany

**Keywords:** Polycystic echinococcosis, Echinococcus oligarthrus, Echinococcus vogeli, Neotropics, South America, metacestode, parasitic emerging disease, historical review

## Abstract

Echinococcosis is a parasitic zoonosis of increasing concern. In 1903, the first cases of human polycystic echinococcosis, a disease resembling alveolar echinococcosis, emerged in Argentina. One of the parasites responsible, *Echinococcus oligarthrus*, had been discovered in its adult strobilar stage before 1850. However, >100 years passed from the first description of the adult parasite to the recognition that this species is responsible for some cases of human neotropical polycystic echinococcosis and the elucidation of the parasite’s life cycle. A second South American species, *E. vogeli*, was described in 1972. Obtaining recognition of the 2 species and establishing their connection to human disease were complicated because the life cycle of tapeworms is complex and comprises different developmental stages in diverse host species. To date, at least 106 human cases have been reported from 12 South and Central American countries.

## Abstract 2

The discovery of an unusual parasitic disease and its causative agents is recounted.

Echinococcosis is a parasitic anthropozoonosis characterized by the development of a larval tapeworm stage (metacestode) in herbivorous intermediate hosts, such as rodents and ungulates, and accidentally in humans. The adult tapeworm is minute and inhabits the small intestine of canids or felids, the definitive hosts. Infections occur in intermediate hosts when they ingest eggs that have been passed in the feces of definitive hosts. In the past, many *Echinococcus* species have been described, but most have been abandoned or reclassified. Molecular phylogeny reconstructions are complex, and the process of taxonomic revision has not yet been completed (*1*). The causative agent of cystic echinococcosis (hydatidosis), the dog tapeworm *E. granulosus* sensu lato, is cosmopolitan. The species responsible for alveolar echinococcosis (AE), the fox tapeworm *E. multilocularis*, is endemic to Holoarctic regions. Recently, *E. shiquicus* n. sp. was discovered in Tibet (*2*). The “neotropical” echinococcal species *E. oligarthrus* and *E. vogel* are confined to the New World. Either species is capable of causing polycystic echinococcosis (PE) in its natural intermediate host and accidentally in humans. Disease due to *E. vogeli* is similar to AE and is characterized by aggressive infiltrative growth and external budding, whereas infection with *E. oligarthrus* has a more benign course. PE thus comprises 2 disease entities. Each is characterized by distinctive epidemiology, clinical manifestations, and morphologic features of the adult and larval parasite (*3*). Today, PE is no longer a medical rarity as more and more cases are being discovered. The prevalence of the disease, however, is unknown.

## First Description of Human Neotropical Echinococcosis

In 1903 and in the years following, Marcelo Viñas in the Buenos Aires province of Argentina described a few cases of what he thought was AE on the American continent. The patients in whom he diagnosed the disease had multilocular cysts with an alveolar aspect, resembling European AE. Notably, the patients came from rural areas and claimed that they had never been out of the country (*4*–*6*). At that time, only *E. granulosus* (described by Batsch in 1786) and *E. multilocularis* were known members of the genus *Echinococcus*. AE had never been detected in South America before and was thought to be restricted to temperate, Holoarctic regions. AE lesions had been recognized as echinococcal 48 years before, in 1855, by Rudolf Virchow (*7*); the causative agent, *E. multilocularis*, had been described by German parasitologist Rudolf Leuckart in 1863 (*8*). The life cycle of the parasite, which involves foxes and rodents, was not elucidated until the 1950s by Robert L. Rausch and Everett L. Schiller (*9*) and Hans Vogel (*10*). Since the patients described by Viñas had never left their home country, he concluded that they must have acquired the disease in Argentina. Would this be the first description of AE in the New World?

## Discovery of Adult *Echinococcus oligarthrus*

Many years earlier, on April 9, 1817, the Austrian emperor, Franz I, had sent a group of natural scientists to Brazil to explore the country. On board one of the ships was 36-year-old Johann Natterer (1781–1843), a passionate ornithologist (*11*). In his past search for parasitic worms in birds, Natterer had studied helminthology at the Naturalien-Cabinete of Vienna’s Hofmuseum under the supervision of Johann Gottfried Bremser (1767–1827), a physician and helminthologist. Natterer was fascinated by Brazil and stayed abroad for 18 years. He explored the area from Rio de Janeiro to Mato Grosso and British Guyana. Natterer returned to Vienna in 1836 with a Brazilian wife, 3 children, and 37 boxes of collected material (*11*). Among the many specimens he brought home was a helminth he had found in the upper part of the small intestine of a puma, *Felis (Puma) concolor*.

**Figure 1:**
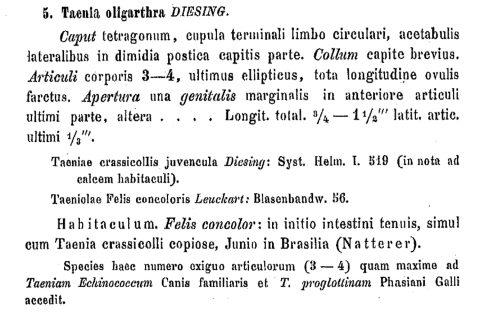
Latin description of adult *Echinococcus oligarthrus* by Karl Moritz Diesing, 1863 ( *14,* p. 370). In addition to the morphologic characterization of the helminth, the 2 prior references from Diesing’s Systema Helminthum (*12*) and from Leuckart’s monography (*13*) are listed. Natterer, who collected the helminth in Brazil, is also mentioned.

Karl Moritz Diesing (1800–1867), a zoologist and successor to Bremser in Vienna, listed the helminth collected by Natterer in his famous Systema Helminthum of 1850 initially under the juvenile form of *Taenia crassicollis* (“*Taeniolae in fele concolore lectae probabiliter pullae*”) found in *F. concolor* (*12*). Rudolf Leuckart (1822–1898) stated in a monograph (*13*) that these helminths may not be seen as juveniles of *T. crassicollis* because they share some characteristics with *T. echinococcus*. Diesing later reclassified Natterer’s specimen as *Taenia oligarthra* in his Revision der Cephalocotyleen, which was presented to the scientific academy in Vienna on November 5, 1863 (*14*). In his Latin description, Diesing noted the presence of only 3–4 proglottids (articuli), hence the name “oligarthrus” (Figure 1). Diesing stated that the low number of proglottids is similar to the number of proglottids in *T. echinococcus*. The organism was still not recognized as an echinococcus, however. The presence of hooks typical for echinococci was not mentioned, and the parasite was placed in a subgroup with hookless tapeworms. All of these scientific descriptions of the South American tapeworm were forgotten by 1903, when Viñas described the cases of possible AE in Argentina.

**Figure 2:**
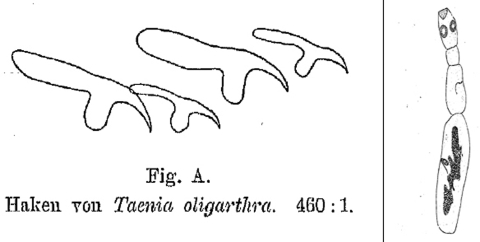
First drawing of the rostellar hooklets (left) and the entire strobilar stage of *Echinococcus oligarthrus* (right). The specimen was listed under no. 396 in the Wiener Hofmuseum. From (*15*).

In 1910, Max Lühe (1870–1916), a German physician and zoologist from Königsberg, requested the cestode material from Vienna and extensively characterized the small helminth. Lühe noted that most of the specimens had lost their rostellar hooks but that they were still present in some organisms (Figure 2). He believed that Diesing must have overlooked the few specimens with hooks. Besides the remarkable difference in body length, no discrepancy with *T. echinococcus* was found. Lühe therefore concluded that *T. oligarthra* and *T. echinococcus* were closely related (*15*). Sixteen years later, Thomas Wright Moir Cameron (1894–1980), from the London School of Hygiene and Tropical Medicine, rediscovered the adult tapeworm in a different South American felid, a jaguarundi (*Felis yaguarondi*), which had died at the London Zoo. Cameron proposed placing *T. oligarthra* in the genus *Echinococcus* (*16*), which had been established by Karl Asmund Rudolphi in 1801. At that time, a cystic larval stage of the parasite had not been found or assigned to a strobilar stage. Whether this parasite could cause human disease was still unknown because no connection to the early Argentinian cases had been established.

## Description of the Larval Stage of *E. oligarthrus*

**Figure 3:**
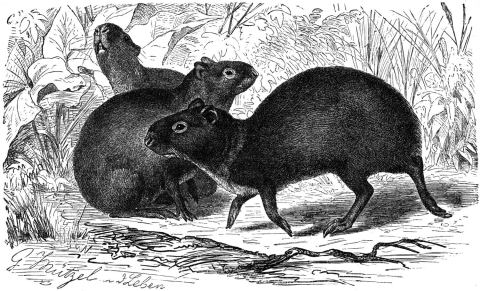
The agouti, *Dasyprocta* sp., one of the natural intermediate hosts for *Echinoccocus oligarthrus*. Drawing by Gustav Mützel (1839–1893).

On May 22, 1914, Emile Brumpt (1877–1951) and Charles Joyeux (1881–1966) from the Laboratoire de Parasitologie in Paris autopsied 4 agoutis (*Dasyprocta agouti*, no. *D. leporina*, Figure 3) in the state of São Paulo, Brazil (*17*). In the spleen and liver of one of these South American rodents they found multiple cysts. The liquid of the cysts resembled hydatid sand. The authors stated that the cuticle of the larva was very thin and that this “reminded us that in *Echinococcus granulosus* this cuticle may reach several millimeters.” The inner surface of the cysts contained a proliferative membrane with many vesicles and protoscolices, the larval stage of tapeworms. The authors extensively described the protoscolices and the amount and shape of the rostellar hooklets they found. They concluded that the cysts in the agouti resembled the general structure of *E. granulosus* cysts. After comparing the hooks with those from *E. granulosus* and *E. multilocularis*, Brumpt and Joyeux concluded that the larva found in the agouti must have originated from a very small tapeworm. They stated that it was “unfortunately impossible to assign our hydatid to a known adult form.” The authors continued to speculate that “due to the origin of the material, it seems absolutely indicated to think of *Taenia oligarthra*.” However, they concluded that the hooklets previously described by Lühe were different in size and shape and that therefore the cysts in the agouti belonged to a not yet described adult tapeworm, which they tentatively named *Echinococcus cruzi*. Their observations were published 10 years later, in 1924 (*17*).

In 1926, Cameron proposed that *E. cruzi* is the larval stage of *E. oligarthrus*, on the basis of the similar size and shape of the rostellar hooks and their origin in the same geographic region (*16*). Cameron had compared the morphologic features of the helminths’ rostellar hooks from the larval stage obtained from the agouti and from the strobilar stage he had rediscovered in the jaguarundi.

## Parasite’s Life Cycle and Human Infection

Around that time, more cases of the emerging South American PE were recorded by Viñas in Argentina (1932, [*18*]). A single case also occurred in Uruguay and was described by Félix Dévé (1872–1951) and co-workers in 1936 (*19*); a second one was described by G. Dardel in 1955 (*20*). Dévé, a French physician, thought that the new South American echinococcosis was a “forme intermédiaire“ between AE and cystic echinococcosis. However, Dévé believed in the unicyst theory of echinococcosis: all types of hydatid disease were caused by a single *Echinococcus* species (*21*,*22*).

In 1966, Vernon E. Thatcher and Octavio E. Sousa from the Gorgas Memorial Laboratory in Panama presented a redescription of adult *E. oligarthrus* on the basis of material from a puma in Panama (*23*). They also implicated humans as possible intermediate hosts, which they deduced from a case report by Sousa and Lombardo Ayala in 1965 (*24*). The latter report described the case of a polycystic, multilocular, hepatic cyst in a native Panamanian; the cyst had characteristics distinct from *E. granulosus* and *E. multilocularis* cysts and was probably caused by a parasite indigenous to the American tropics. The authors concluded that the human hydatid possibly represented *E. oligarthrus*. They further suggested that the polycystic multilocular human hydatidosis of the Panama-Colombia area, studied around that time by Antonio D’Alessandro from the Tulane University International Center for Medical Research in Colombia, might be caused by the same species of parasite.

One year later, adult *E. oligarthrus* was found again by the same authors in the small intestine of another wild felid, the Panamanian jaguar (*Felis* [*Panthera*] *onca*) (*25*). After a reexamination of material previously misconstrued by others, Thatcher and Sousa concluded that a metacestode found in a nutria (*Myocastor coypus*), a South American rodent that had died in a United States zoo, was the larval stage of *E. oligarthrus* (*26*). Until then, various South and Central American felids had been considered to be definitive hosts of *E. oligarthrus,* and the presumed larval stage of the parasite had been discovered in rodents from the same geographic area. Experimental work was needed at that time to elucidate the biologic definition and the life cycle of the parasite. Proof had to be found that the formerly described *E. cruzi* was indeed the presumed metacestode stage of *E. oligarthrus*.

Sousa and Thatcher achieved this aim in 1969 by experimentally inducing hydatidosis in different rodent species. Among others, climbing rats, spiny rats, and agoutis were fed gravid proglottids of *E. oligarthrus* obtained from a naturally infected puma (*27*). In these successfully infected intermediate hosts, mature metacestodes showing similar morphologic features to *E. cruzi* developed in the muscles and inner organs. In a second experiment, the experimentally induced hydatids of the agoutis transformed into adult and mature *E. oligarthrus* in the feline intestine when fed to domestic cats. In return, parasite material obtained from the infected cats produced hydatid cysts in agoutis. In contrast, dogs could not be infected. The house cat was therefore implicated as playing an important role as definitive host and as a potential risk to humans. The life cycle of the parasite, however, was considered to be mainly sylvatic (*27*). After nearly 120 years, the mystery of human PE seemed finally solved. In 1972, however, a second South American species, *E. vogeli*, was discovered.

### Discovery of a Second South American Species, *E. vogeli*

In late 1969 or early 1970, Martin Stummer, an animal dealer at Amazon Ltd, a company supplying animals for zoos, captured a bush dog (*Speothos venaticus*) in the province of Esmeraldas in Ecuador. The animal was sent to the Los Angeles Zoo and routinely examined. After a deworming treatment had resulted in the expulsion of numerous cestodes of the genus *Echinococcus*, Calvin Schwabe from the School of Veterinary Medicine in Davis, California, examined the helminths and found unusual morphologic characteristics. Robert L. Rausch from the Arctic Health Research Center in Fairbanks, Alaska, and J. J. Bernstein from Venice, California, described a small helminth, which differed substantially from all other recognized species of *Echinococcus* (*28*). They named the species *E. vogeli* in recognition of Hans Vogel (1900–1980) from the Bernhard-Nocht-Institute in Hamburg, who contributed to the elucidation of the life cycle of *E. multilocularis*. On the basis of the morphology of the rostellar hooks and other characteristics, Rausch and Bernstein were able to describe this new species in 1972. In the same year, Thatcher concluded that *E. oligarthrus* was likely the cause of all cases of human and animal PE in the neotropics (*29*). However, with the description of a new indigenous species, uncertainties arose about the etiologic role of *E. oligarthrus* in PE (*30*). None of the researchers could know at that time that *E. vogeli* would soon be the most frequently encountered species of the 2 indigenous South American echinococcal tapeworms.

The synonymy of *E. cruzi* with *E. oligarthrus* was then questioned. A reexamination in 1984 of material obtained from Brumpt’s and Joyeux’ initial case of the agouti demonstrated that the larval stage of *E. oligarthrus* was indeed the causative organism (*31*). In contrast, the metacestode found in the nutria and in the Panamanian patient described in 1965 was shown to be *E. vogeli* (*30*,*32*). The 11 cases described by Viñas in Buenos Aires and those noted by Dévé and Dardel from Uruguay could not be definitively assigned to either *E. oligarthrus* or *E. vogeli*. The presence of protoscolex hooklets, which are used for discrimination, was not described in detail in these reports (*33*). However, the cases are most likely caused by *E. oligarthrus* because the final host of *E. vogeli* is not found in those areas (*33*). By the end of 2007, 3 cases of proven *E. oligarthrus* infection in humans have been reported: 1 cardiac case from Brazil (*34*) and 1 orbital case each from Suriname (*35*) and Venezuela (*36*).

**Figure 4:**
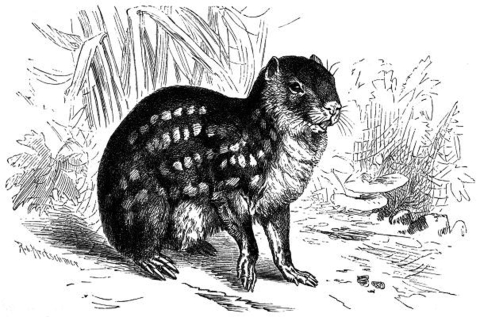
The paca, *Cuniculus paca*, the natural intermediate host for *Echinococcus vogeli* and rarely *E. oligarthrus*. Drawing by Robert Kretschmer (1818–1872).

Rausch and Bernstein predicted, on the basis of the known predator-prey relationship of the bush dog, that the larval stage of *E. vogeli* would also occur in rodents, including pacas (*28*). Indeed, parasitic cysts were found in a Colombian paca (*Cuniculus paca*, Figure 4) in 1975. The material was experimentally fed to a dog; in addition, larvae obtained from a Colombian human patient with PE (*37*) were given to a second canid. From both dogs, the strobilar stage of *E. vogeli* was later recovered (*30*). As sufficient material was collected from the field in Colombia and obtained from experimentally infected animals, R.L. Rausch, V.R. Rausch, and A. D’Alessandro were able to morphologically distinguish *E. vogeli* from *E. oligarthrus*. The rostellar hooks of each of the 2 South American species were found to consistently differ in length and form, which permitted discrimination of the tapeworms’ larval stages. As a consequence, known human and animal cases of PE were reexamined, and some cases thought to have been caused by *E. oligarthrus* were shown to have been caused by *E. vogeli* instead (*32*). *E. vogeli* typically has a thick laminated outer layer and a thin inner germinal layer, whereas *E. oligarthrus* has the reverse. Calcareous corpuscles are abundant in the germinal layer and in the protoscolices of *E. oligarthrus* but are almost absent in *E. vogeli* (*33*).

In just a few years, a second indigenous South American echinococcal species had been discovered, and the life cycle of the parasite, involving the bush dog and the paca, had been described. In a survey of Colombian mammals, 73 (22.5%) of 325 pacas harbored metacestodes of *E. vogeli*, but only 3 (0.9%) of pacas harbored *E. oligarthrus*. Twenty (6.2%) more pacas were shown to be infected with polycystic larvae, but the species involved could not be determined. In addition to the bush dog, a domestic dog belonging to a hunter was found to be naturally infected with adult *E. vogeli* (*38*). Researchers then assumed that domestic dogs might play a role in the transmission of parasite eggs to humans.

## Current Situation

As of 2007, at least 106 human cases of PE from 12 countries have been documented. The disease occurs exclusively in rural areas of the American tropics and often in regions where *E. granulosus* is not present (*33*). Most cases are reported from Brazil and Colombia (*33*,*39*), but PE is endemic from Nicaragua to Chile (*35*). Its rising frequency (12 cases from 4 countries in 1979, 72 cases by 1997, and 86 cases from 11 countries as of 1998) shows that human PE is an emerging disease and no longer a medical curiosity (*33*). Most cases are caused by *E. vogeli*, but many cases could not be assigned specifically to any of the 2 South American echinococcal species because the presence of hooks was not reported (*33*,*39*). In an advanced laboratory setting, *Echinococcus* species can be distinguished by PCR followed by sequencing or restriction fragment length polymorphism analysis (*40*). Parasite material obtained from those infected, for whom a diagnosis cannot be made by means of classic parasitology, can now be subjected to methods of molecular biology. Why most PE is caused by *E. vogeli* is unclear. Some have speculated that because felids cover their feces, contact with infectious ova of *E. oligarthrus* is less likely than contact with eggs of canid-borne *E. vogeli* (*33*). Accordingly, similar proportions in infection rates of the respective natural intermediate hosts have been found (*38*). Seven species of wild felids that were naturally infected with *E. oligarthrus* have been found. The geographic distribution of wild cats extends from northern North America to southern Argentina. In contrast, the bush dog, the only natural definitive host for *E. vogeli*, is found from Panama to south Brazil. The published number of human cases is probably just the tip of the iceberg (*33*); the true prevalence of human PE is far from being known.
